# The rising dominance of microbiology: what to expect in the next 15 years?

**DOI:** 10.1111/1751-7915.13953

**Published:** 2021-10-29

**Authors:** Roshan Kumar, Utkarsh Sood, Jasvinder Kaur, Shailly Anand, Vipin Gupta, Kishor Sureshbhai Patil, Rup Lal

**Affiliations:** ^1^ Post‐Graduate Department of Zoology Magadh University Bodh Gaya Bihar 824234 India; ^2^ The Energy and Resources Institute Darbari Seth Block, IHC Complex, Lodhi Road New Delhi 110003 India; ^3^ Department of Zoology Gargi College University of Delhi Siri Fort Road New Delhi 110049 India; ^4^ Department of Zoology Deen Dayal Upadhyaya College University of Delhi Dwarka New Delhi 110078 India; ^5^ Indira Paryavaran Bhawan Ministry of Environment, Forest and Climate Change Lodi Colony New Delhi 110003 India; ^6^ Department of Biological Sciences P. D. Patel Institute of Applied Sciences Charotar University of Science and Technology (CHARUSAT) Changa Gujarat 388421 India

## Abstract

What microbiology beholds after a decade and a half in the future requires a vision based on the facts and ongoing trends in research and technological advancements. While the latter, assisted by microbial dark matter, presents a greater potential of creating an upsurge in in‐situ and ex‐situ rapid microbial detection techniques, this anticipated change will also set forth a revolution in microbial cultivation and diversity analyses. The availability of a microbial genetic toolbox at the expanse will help complement the current understanding of the microbiome and assist in real‐time monitoring of the dynamics for detecting the health status of the host with utmost precision. Alongside, in light of the emerging infectious diseases, antimicrobial resistance (AMR) and social demands for safer and better health care alternatives, microbiology laboratories are prospected to drift in terms of the volume and nature of research and outcomes. With today’s microbiological lens, one can predict with certainty that in the years to come, microbes will play a significant role in therapeutic treatment and the designing of novel diagnostic techniques. Another area where the scope of microbial application seems to be promising is the use of novel probiotics as a method to offer health benefits whilst promoting metabolic outputs specific for microbiome replenishment. Nonetheless, the evolution of extraterrestrial microbes or the adaptation of earth microbes as extraterrestrial residents are also yet another prominent microbial event one may witness in the upcoming years. But like the two sides of the coin, there is also an urgent need to dampen the bloom of urbanization, overpopulation and global trade and adopting sustainable approaches to control the recurrence of epidemics and pandemics.

## Graphical abstract

Microbial research could lead to the development of in‐situ and ex‐situ rapid microbial detection systems based on microbial dark matter in the next 15 years. As a result of this anticipated shift, microbial cultivation will rapidly rise, altering the diversity of microorganisms and underlying lineages. We will be able to better comprehend the microbiome in real time and the health condition of the host as a result of being able to expand the microbial genetic repertoire. Similarly, due to developing infectious illnesses, antibiotic resistance, and social norms for safer and better health care options, the volume and nature of activities required of microbiology laboratories will change.

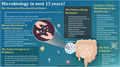

## Introduction

In recent years, microbiome research has emerged as an integral component of all simple and complex ecosystems. The dynamism of microbial diversity has closely influenced the resilience of all life forms. Almost four centuries have been consumed to comprehend the remarkable influence of microorganisms on the host system after the first documentation of microbes by Antoine van Leeuwenhoek in the 17th century (Gest, 2004). Interestingly, in the last two decades, the insight into the microbial world has dramatically improved due to the recent advancements in science and technology. Over the years, microscopy and sequencing have emerged as two dominating technologies that have contributed to a farther expanse of microbiology as a discipline. These have enabled the human‐kind to *see the unseen with profound depth*. Emphasizing more on sequencing, the cost of data generation and interpretation has dropped significantly leading to a massive amount of data in public repositories. For instance, it took almost 14 years (1990–2003) to completely sequence the first human genome, but a short span of slightly over a decade was sufficient to generate an integrated map of 2504 human genomes displaying structural differences (Sudmant *et al*., 2015). The cost of sequencing the first human genome was $3 billion (Hood and Rowen, 2013), but we now hope to sequence human genome for less than $100 (Singh, 2018).

Given the vast new realm of microbiome research, we intend to emphasize prospective developments in microbiome research in the near future. To summarize the important advances in microbiome research and their future implications, this review tries to stitch in six key areas spanning: (i) The outreach of microbial dark matter in next decade; (ii) The future of drug resistance and its probable solution; (iii) Human microbiome: a promising solution in the coming decade; (iv) The future prospects of probiotics and (v) Frontiers of space microbiome in the next decade and (vi) The next pandemic we may expect.

## The outreach of microbial dark matter in the next decade

The inception of the word dark matter in microbial context was proposed to infer the existence of the vast majority of microbes which has not been axenically cultured and at the same time, their existence is supported by metagenomic gene inventories (Marcy *et al*., 2007). Prof. Julian Davis, University of British Columbia quoted that ‘Once the diversity of the microbial world is catalogued, it will make astronomy look like a pitiful science’. Therefore, the future prospects lie in the expansion of the realms of microbiology to find newer ways to unfold the full phylogenetic diversity of microbes and their immense applied significance. Currently, there are few approaches, which are being implemented to characterize these microbes. To begin with isolation, sorting of single cells or colonies, we implement methods such as fluorescence‐activated cell sorting (FACS) (Rinke *et al*., 2014), serial dilution, micromanipulation (Woyke *et al*., 2010), optofluidics (Landry *et al*., 2013) and laser‐capture microdissection of tissue samples (Frumkin *et al*., 2008). Once the cells are separated, to make their DNA accessible, cells are lysed using methods such as alkaline lysis (Clingenpeel *et al*., 2014). This is followed by whole genome amplification using techniques such as isothermal multiple displacement amplification (MDA) or multiple annealing and looping‐based amplification Cycles (MALBAC) to obtain sufficient material for subsequent data generation from a single cell leading to the identification of microbial dark matter across a diversity of environmental samples (Zong *et al*., 2012).

In recent years, the term ‘culture‐omics’ has been coined to describe the process of creating a microbial library employing robotic liquid handling and a variety of culture conditions and nutrient media, followed by identification using MALDI‐TOF mass spectrometry and 16S rRNA gene sequencing (Lagier *et al*., 2015, 2018). This technique has been extensively employed to culture the human‐associated microbes. Of note, by the year 2015, a total of 2172 microbial species were cultured from human samples at least once (Hugon *et al*., 2015), which saw a spur of 28% (*n* = 2776) by 2018 (Lagier *et al*., 2018) and by the year 2020, a 17% increase was observed by adding 477 species (Diakite and Dubourg, 2021). Between 2018 and 2020, 63% of the total species were cultured using the culturomics technique (Diakite *et al*., 2021) increasing the count to 3253 bacterial species which have been cultured at least once from human samples (Diakite *et al*., 2021). Similar to culturomics, many high throughput methods are being developed to uncover microbial dark matter. For instance, Zou *et al*. (2019) cultured 6487 bacterial isolates from the faeces of 155 healthy donors and reported 338 species‐level clusters (ANI ≥ 95%) corresponding to 1520 genomes (Zou *et al*., 2019). Interestingly, among 338 clusters, 134 unique clusters (corresponding to 264 genomes) were reported for which there was no reference genomes in the NCBI database, while 50 clusters were so unique that they did not fall within any sequenced genera (Zou *et al*., 2019).

Further, to bridge the gap between microbial dark matter and their cultivation, massive metagenomics sequencing is being implemented to facilitate the genomic information of isolation‐recalcitrant members of the community. For instance, a recent study extensively explored the human microbiome diversity by reconstructing 154 723 microbial genomic bins from 9428 metagenomic datasets (Pasolli *et al*., 2019). By doing so, they recapitulated 4930 species‐level genomic bins among them for 77% (*n* = 3796) genomes, there was no reference available in the public depositories (Pasolli *et al*., 2019). Even today, when the sequencing data generation has reached an exemplary status, a variety of outcomes remain to be explored, and thus, these studies re‐emphasize the unexplored phylogenetic and functional diversity of microbial dark matter, allowing researchers to identify previously undetected microorganisms or microbial mechanisms unique to a population or environment. Because it is difficult to recreate natural conditions in the lab to accommodate new microorganisms, attempts are being made to culture bacteria in their native surroundings. Bollmann and Lewis (2007) devised a diffusion‐chamber method to culture the isolates from the marine sediment environments and reported the isolation of species from rarely cultivated groups (Bollmann *et al*., 2007). This device was a combination of polycarbonate membrane of 0.03 µm pore size attached to the bottom of a stainless‐steel ring and the inner space filled with microbial cells mixed with agar for *in‐situ* microbial cultivation of the target microorganisms. This technique led to the isolation of bacterial species from rarely cultivated groups, namely, *Spirochaetes*, *Verrucomicrobia*, *Deltaproteobacteria* and *Acidobacteria*.

Berdy *et al*. (2017) developed a cultivation platform known as isolation chip (iChip), made up of three elements, a 96 through‐holes plastic plate, two rectangular polycarbonate membranes with pore size 0.03 µm and silicon glue (Berdy *et al*., 2017). The deployment and assembly of iChip is relatively a simple protocol and can be performed easily. Once the environmental sample is collected, after serial dilution, the cells are loaded onto the iChip, then the chip is incubated in the natural environment to access the naturally occurring nutrients and growth factors (Berdy *et al*., 2017). This is followed by the retrieval and domestication of colonies from the iChip to the laboratory. This approach has improved the microbial recovery from 5 to 300 folds, depending on the sample type and environment (Berdy *et al*., 2017).

Going one step further ahead in inventing novel cultivation strategies, Prof. Slava Epstein and his team at Northeastern University devised a nanofluidic device that can not only autonomously isolate individual microbial cells but also functionally characterize these microbial cells (US10793891B2).

Keeping in mind, the recent advancement in the field of microbial dark matter and the prevailing perception of microbiologists, in the next 15 years we should witness:

1. Advancement in the existing tools and techniques of cultivation, which will include minimum handling and maximum automation.
2. Newer methods to study the microbial interactions and their role in cultivation both in *ex‐situ* and *in‐situ* environments.
3. Cultivation of ‘missing’ species of microbes, leading to the spur in microbial cultivation representing novel lineages and previously uncultured clades, which will revolutionize the view of microbial evolution and diversity.
4. The discovery of microbial dark matter will further unveil new processes/phenomena leading to theoretical advancements and ecological understanding from those already described.
5. Additionally, the discovery will lead to the identification and characterization of novel compounds and their biosynthetic gene clusters with potential applications in health and medicine.

## The future of drug resistance and its probable solution

The misuse, abuse or overuse of antibiotics has led to serious repercussions on the health of mankind due to the generation of microbes that are resistant to a spectrum of antibiotics. Bacterial isolates show a different level of susceptibility towards the available antibiotics and are termed as multidrug‐resistant (MDR), extensively drug‐resistant (XDR) or pan drug‐resistant (PDR). MDR isolates show acquired non‐susceptibility to at least one agent in three or more antimicrobial categories, XDRs remain susceptible to only one or two categories of anti‐microbials and PDR is as non‐susceptible to all agents in all antimicrobial categories (Magiorakos *et al*., 2012). This problem is very serious nowadays and is referred to as antimicrobial resistance (AMR). According to World Health Organization (WHO), drug‐resistant diseases have claimed 700 000 lives worldwide and this number is projected to reach 10 million by 2050 (*New report calls for urgent action to avert antimicrobial resistance crisis*, 29 April 2019, WHO). Microbiology has a very important role in understanding the problem of AMR and finding probable solutions. The next 15 years will surely be crucial, where the understanding of microbiology in society will help in reducing this menace.

Although resistance to antibiotic drugs can occur naturally due to random mutations in the genetic code of microbes, the main reason for the development of AMR nowadays is not taking the proper dosage of antibiotics or consuming antibiotics for the wrong reasons. The major implication is that now some bacteria are resistant to all the antibiotics that are being used in clinical practices. Pathogenic infections like tuberculosis, pneumonia and salmonellosis are becoming harder to treat leading to higher medical costs, longer hospital stays and an increase in death of individuals worldwide (Prestinaci and Pezzotti, 2015). The problem is not specific to any particular age group or country, however, in places where antibiotics can be bought without a prescription, the emergence and spread are lethal (Llor and Bjerrum, 2014). Therefore, there is an urgent need to rectify how antibiotics are prescribed and used worldwide. Even if efforts are made to produce newer medications and regulate the status and problem of AMR, without a change in human behaviour, all of this will go in vain. The behavioural changes include opting for vaccination, good food hygiene, hand‐washing and practicing safer sex, taking antibiotics only with a proper prescription in case of any infection (Okeke and Lamikanra, 1999).

The crisis of antibiotic resistance is extremely serious as without antibiotics the death toll due to infectious diseases will increase several times and conditions will be similar to the pre‐antibiotic era (Ventola, 2015). Humans and bacteria have been at odds for centuries, and we must acknowledge that we can never win a war against microorganisms since microbes allow us to survive. Antibiotic resistance has been extensively studied in a group of bacteria known as the ESKAPE pathogens. The next section deals with the details about ESKAPE pathogens and also the mechanisms of AMR generation that are crucial for developing probable solutions.

### ESKAPE pathogens and mechanisms of antimicrobial resistance generation

The deadliest pathogenic microbes that can escape our immune response as well as those which are equipped with rapidly growing multi‐drug resistant properties are termed ESKAPE pathogens. The acronym is used for microbial species from *Enterococcus faecium*, *Staphylococcus aureus*, *Klebsiella pneumoniae*, *Acinetobacter baumannii*, *Pseudomonas aeruginosa* and *Enterobacter* species are the leading cause of pathogenic infections in the nosocomial setting with MDR (Santajit and Indrawattana, 2016). These pathogens are designated at the priority status by WHO and are therefore suitable for studying this problem and also applying probable solutions.

### Mechanism of development of antibiotic resistance

The most common way for the development of antibiotic resistance is the generation of genetic variants that can either metabolize the antibiotic or throw it out of the bacterial cells through genetic mutation under the pressure of drugs. The problem becomes more severe when these gene variants are transferred horizontally among other bacteria by horizontal gene transfer. ESKAPE pathogens have developed resistance mechanisms against lipopeptides, macrolides, oxazolidinones, fluoroquinolones, β‐lactams, tetracyclines, β‐lactam–β‐lactamase inhibitor combinations and antibiotics that are the last line of defence, including glycopeptides, carbapenems and clinically unfavourable polymyxins (De Oliveira *et al*., 2020). It has been understood how these microbes have become non‐susceptible to antibiotics (De Oliveira *et al*., 2020). Polymyxins being toxic will likely be replaced and two novel antimicrobial agents’ cefiderocol and eravacycline have been recently approved for treating difficult‐to‐treat *A. baumannii* infections (Bassetti *et al*., 2021). Microbes deactivate or modify the antimicrobial compounds by producing enzymes like β‐lactamases and aminoglycoside‐modifying enzymes. In summary, there are three fundamental mechanisms by which resistance against antimicrobial is gained (i) enzymatic degradation of antimicrobials (ii) structural changes in the target proteins of microbes and (iii) alteration in membrane permeability of microbes towards antibiotics (Dever and Dermody, 1991).

A recent study has shown the effect of light on important pathogenic determinants in ESKAPE pathogens. Light has shown to have broad spectrum antimicrobial effect (Gwynne and Gallagher, 2018). Light has been shown to reduce the virulence in *Pseudomonas aeruginosa* and *Acinetobacter nosocomialis*. However, it has been linked with increase virulence in *Staphylococcus aureus* and *Acinetobacter baumannii* (Tuttobene *et al*., 2021). This shows the adaptive capabilities of microbes towards different antimicrobials and no solution against the ever evolving pathogenic microbes is a permanent solution.

### Probable solutions of AMR: What to expect in the next 15 years

#### Preventing infection and spread of resistance: the role of government and regulatory agencies

Tracking the spread of resistant pathogens in the human population will be an extremely important parameter. The regulatory agencies and governments must have a global surveillance system for AMR. The idea of the Global Antimicrobial Resistance Surveillance System (GLASS) was put forward by WHO recently in 2014. It aimed to appropriately scale and monitor the general situation worldwide (Kajihara *et al*., 2020). Hospitals are AMR hotspots where pathogens can persist for years in the sewage, plumbing and hospital surfaces. Metagenomics studies can be carried out on regular basis to determine the situation of AMR in hospital settings. This data should be catalogued and deposited in dedicated repositories that can be used to study the type of microbes thriving in the hospitals and the presence of antibiotic resistance genes. Temporal collection of the data will also be used in near future to trace the evolutionary changes occurring in the microbial communities and scientist can then plan in advance their strategies to curb the spread of resistant microbes.

Another important aspect will be promoting proper hygiene among individuals where spread of infectious diseases is extremely high. The recent coronavirus pandemic is a prime example, where maintaining proper hygiene can limit the spread of the infection, similarly in case of microbial infection if the rate of infection is low than the problem of AMR will be in check.

#### Improving antibiotic prescribing/stewardship

Over the counter sales of antibiotics need to stop as soon as possible. Proper prescriptions from registered doctors must be presented to the pharmacist for obtaining antibiotics. Another problem is that people do not complete the required course of antibiotic medication. Microbial literacy for society will play an extremely important role by making the individuals understand how these antibiotics work and the importance of completing the course of medication.

##### Phage therapy

Bacteriophages are viruses that kill specific bacteria. These phages are nowadays used to lower the load of pathogenic bacterial infections. It is one of the most widely accepted alternatives or a supplement to treat serious cases of AMR. Now attempts are being made worldwide for developing phage therapy for a number of bacterial infections that have high incidence of drug‐resistant infections. These phages are extremely selective towards their host and this specificity is both advantageous and disadvantageous. It is still difficult to find the exact phage and dosage that is needed against a particular strain of infectious bacteria because of the strain specificity towards the host. Therefore, attempts are now days focussed on developing a phage cocktail having phages with specificity towards different strain of pathogenic bacteria. Although no serious effect on human health has been reported till now, still many considerations have to be taken into account as the effect of interactions between phage, bacteria and human is still unknown (Lin and Koskella, 2017). A recent study has shown that there is no effect on the normal microbiome of the patients receiving phage therapy hence it can be considered as a safe alternative to reduce the pathogenic load of microbes with AMR (Mulani *et al*., 2019).

The future of phage therapy is in the development of personalized cocktails of bacteriophages based on the microbiome profile of the infected human. This will surely reduce the incidence of drug resistance and also keep in check the spread of AMR in bacteria.

##### Developing new drugs, combinatorial therapy, Repurposing exiting drugs and diagnostic tests

Alternative therapies such as the use of antibiotics in combination or with adjuvants, bacteriophages, nanoparticles, antimicrobial peptides and photodynamic light therapy are widely reported (Mulani *et al*., 2019; De Oliveira *et al*., 2020). Newer drugs like durlobactam/sulbactam are in phase 3 of clinical development for tacking the resistant strains of bacteria (Bassetti *et al*., 2021). Antibiotic combination therapy is a futuristic approach where the known antibiotics are used in different combinations to have improved effects. Although this technique looks promising, there were almost no experimental methods to determine the efficacy and effects of combination therapy, however, recently an approach was designed to test the combination therapy under realistic epidemiological conditions in vitro using a robotic liquid handling platform (Angst *et al*., 2021). The results seem to be promising as the result indicates combination therapy is more effective than monotherapy (Angst *et al*., 2021).

Drug repurposing that is identifying the use of already available drugs for therapeutic uses against pathogenic organisms for which it has not been used earlier. Since development of a new drug is difficult and cost intensive, repurposing anthelmintic, anti‐cancer, anti‐inflammatory and immunomodulatory, antipsychotic and antidepressant and other classes of drugs are attempted to treat infections caused by bacteria and fungi (Miro‐Canturri *et al*., 2019). This holds promising aspect for in the next decade and more information from the microbiological pathways can help to re‐purpose the existing drugs against resistant microbes. In addition to these, microbial marker/microbiome profiling will also be taken up for diagnostics of different diseases and development of personalized medicine/ therapies.

##### Targeting the quorum sensing of pathogenic bacteria

The pathogenic bacteria use quorum‐sensing mechanism mediated by signal molecules to regulate the expression of virulence genes required for biofilm and toxin formation. Quorum‐sensing inhibitors are anticipated to be one of the best substitutes to antibiotics (Zhao and Yu, 2020). Newer advancement in microbiology will help us understand the mechanism of quorum sensing and this information will be used to quench the quorum sensing of pathogenic bacteria.

## Human Microbiome: a promising solution in coming decade

Among all major aspects of microbiology in relation to human health, microbiome‐assisted research has gained most of the coverage in last decade and it is believed that it will go further for next century without any fall (Ravel *et al*., 2014; Ma *et al*., 2018). The human gut microbiome has been presented as a potential regulator of wellness and diseases; therefore, the coming years in microbiology will be replete with medicinal discoveries of microbiome studies (Kho and Lal, 2018). With the rising economical share of therapeutics world‐wide, window of personal medicine based on individual gut microbiome has also widened slightly (Ihekweazu and Versalovic, 2018). Earlier in 1970s, the predicted microbial cell to human cell ratio was 10 : 1 (Luckey, 1972), until 2016 when (Sender and Fuchs, 2016) re‐quantified the exact ratio to be 1 : 1 with great variation (15–60%) existing among individuals. More importantly, it is not just the number but the functional contribution provided by these bacterial communities to human physiology which is observed as host microbiome drug response during any treatment. Since last decade, microbiome has marked its potential role in promising medical therapeutics solutions such as Fecal Microbiota Transplant (FMT) for recurrent *Clostridium difficile* infections (rCDI), pre/pro/post‐biotic therapies for modulating host response and most recently in pharmo‐microbiomics for differential drug response among hosts (Sharma *et al*., 2020).

The most promising microbiome‐applied solution that has been scientifically validated and approved by FDA (with consent of regulatory authorities) in many countries for rCDI patients is FMT (Davidovics *et al*., 2019). Because rCDI is a life‐threatening disease with limited patient care, administering a suitable/healthy FMT to the recipient significantly eradicates and lowers AMR situations. The successful cases of FMT have been found stable to a longer duration then expected (up to 1 year) and many patients recovered completely (Millan *et al*., 2016). FMT's results have been found to vary with certain illnesses such as IBD, with nearly 60% of younger patients experiencing remissions in a trial of 122 IBD patients (Colman and Rubin, 2014). In such cases, the FMT has been now improved where application of sterile faecal filtrate, without bacterial compositions, only DNA, microbial debris, metabolites, viruses were used. This method resulted in much better conditions where all the subjects were recovered from rCDI up to a longer duration (Ott *et al*., 2017). FMT's success is entirely dependent on the donor's faecal microbial diversity, leading to the concept of the Super Donor, the most optimal donor for the host, taking into account significant aspects such as ethnicity, genetics, races, pathogen‐free excrement and immune responses (Kump *et al*., 2018). Clinically, certain IBD cases where donors were found rich in *Clostridium* clusters IV and XIV (*Oscillibacter*, *Dorea*, *Roseburia* and *Blautia*), the bacterial composition had stable results (Vermeire *et al*., 2016). Even though FMT is one of the most revolutionizing microbial therapy for rCDI, but it still has limitations and must be used cautiously as there are unpredictable results such as obesity and diabetes (Kelly *et al*., 2015). This has evolved into an industrial‐grade concept, with ‘OpenBiom’, a non‐profit company, being established to screen ‘Super Donors’, with each successful donor receiving a $50 donation (https://www.openbiome.org/).

Apart from FMT, human microbiome studies have also recently contributed much in elucidating the modification of chemical structures of drugs by microbial enzyme that were being administered to the patients of physiological diseases like circulatory system and gut infections (Hall *et al*., 1999). This majorly includes the common drugs such as omeprazole, ibuprofen, acetaminophen, sorivudine, nitrazepam and lovastatin. A known cardiovascular drug like digoxin was found deactivated or attenuated by cardiac glycoside reductase present in *Eggerthella lenta,* a gut resident known from decades (Haiser *et al*., 2013). Likewise, *Bacteroides* spp.; *B. ovatus* and *B. thetaiotaomicron* were found increasing the drug toxicity effect for sorivudine, a drug used for treating viral infections (Nakayama *et al*., 1997). Similarly, the few of the aerobic bacteria spp. including spp. of *Bacteroides* of human gut were found to be metabolizing an antibiotic called omeprazole (Watanabe *et al*., 1995). This drug is most commonly used for treating acid refluxes related to stomach and oesophagus. Microbial enzyme activity reduces the efficacy of omeprazole. Pharmacomicrobiomics has entered the leading mortality disease architecture‐Cancer (Nichols and Peters, 2019). It was found that during the chemotherapy session a drug called irinotecan (CPT‐11) was interacting with host cytochrome P540) enzymes, and downregulation of SN‐38 by UDP‐glucronosyltransferase enzymes (de Man *et al*., 2018). CPT‐11/SN‐38 treatment were linked with diarrhoea and were thought be manipulated with inhibitors for UDP‐glucuronidase and were found highly effective (Innocenti *et al*., 2004; de Man *et al*., 2018). Recently, framework was proposed by Alexender *et al*. to cover microbial metabolism action for therapeutic drugs in use, TIMER ‘Translocation, Immunomodulation, Metabolism, Enzymatic degradation, Reduces diversity & ecological variations’ (Alexander *et al*., 2017). This framework has now changed the way of administration of any common drug with possible effect by gut microbial community (Alexander *et al*., 2017).

Thus, with known complexity, the microbial paradigm is shifting with a combination of reductionist and integrated system level approach to increase the benefits with pharmacological drugs. This has opened new avenues for alleviating drugs’ effect and more effective care under certain disease circumstances and will become more comprehensive with increasing knowledge of microbiome in coming years. Data integration of metagenomics, meta‐transcriptomics and metabolomics in pharmaceutical industries with amalgamation of advance and modern computational approach like AI and Machine learning could be a valuable resource in coming future for mankind. The direction of microbiome research headed towards the estimation of presence or absence of bacterial strains in the microbiome using radio frequency identification (RFID) signal fluctuations (Cullen *et al*., 2020). In the next 15 years, we may witness the technologies cum analytical methods to test the real‐time microbial markers and characterize the onset or progression of various microbial diseases.

## The future prospects of probiotics

Literally meaning ‘for life’, the history of probiotics nearly dates back to the origin of humans (Gasbarrini and Bonvicini, 2016). Ancient scriptures including the Ayurveda describes the benefits of milk and its products for human health (Ozen and Dinleyici, 2015). Excavation studies of the by‐gone civilizations especially the Mesopotamian and Egyptian have also provided sufficient evidence of using dairy products then. The use of fermented milk and its products, mushrooms, soy sauce, wine, cheese, pickled vegetables and many more can be traced back to 1000 to 10 000 BC. Thus, the concept of probiotics existed much prior to the discovery of bacteria. But its modern history is just over a century old when Metchnikoff attempted to relate the role of microorganisms with human health. Since then, this field has witnessed a growing attention of researchers worldwide. Recent advances in microbiome research and an enhanced public vocabulary of the gut microbiota has further fetched much attention towards probiotics (defined as ‘*live microorganisms that when taken in sufficient amounts can provide health benefits*’ (Gibson *et al*., 2017), prebiotics (‘*a substrate that is selectively utilized by host microorganisms conferring a health benefit*’ (Gibson *et al*., 2017)) and also synbiotics (blends of probiotic and prebiotic together). With a surge in metagenomics studies and high‐throughput analytical tools, probiotics have taken a centre stage for researchers across the globe. There has been an increased focus on identification and characterization of probiotic candidates, their maintenance and potency of withstanding the acidic gut environments (Antony, 2018), the intricate details of their mode of action in the host ecosystem, their ability to colonize the GI tract and in uncovering their specific influence on the host. Alongside efforts are also being put to devise *in‐vitro, in‐vivo* and *in‐silico* models for genetic characterization and manipulation of potent strains and to unravel the functionality of probiotics candidates in the host system (Spacova *et al*., 2020). Though comprehensive studies supported with systemic analyses during the last couple of decades have provided insights into the positive role of microbiota in diverse life‐sustaining processes including digestion, immunity, bowel functions (Vandeputte *et al*., 2017), bone mineralization (Abrams *et al*., 2005), mental well‐being (Belkaid and Hand, 2014) and preventing the risk of respiratory infections (Hatakka *et al*., 2001), vaginal dysbiosis (Reid *et al*., 2003), allergies (Dang *et al*., 2013; Cuello‐Garcia *et al*., 2015), cardiovascular diseases (Antony, 2018), obesity (Cunningham *et al*., 2021) and diarrhoea (Guandalini, 2011) to name a few; the precise relationships and co‐dependencies (Spacova *et al*., 2020) remain quite blurred.

Working as allies with the gut microbiota, probiotics have been well documented in not only modifying the host immune system but also in modulating the metabolic response and the physiology of the organs (Mikelsaar *et al*., 2016; Upadrasta and Madempudi, 2016). As a consequence, they are looked upon with a promising future especially in preventive healthcare. An uphill trend of consumer awareness primarily owing to increased literacy, rise in the standard of living and also the access to information on the click of fingertips is driving the global probiotics and prebiotics on a high scale. It is anticipated that in less than half a decade from now, the global market of probiotics and prebiotics shall expand by a compound annual growth rate (CAGR) of 6.9% (https://www.grandviewresearch.com/industry‐analysis/probiotics‐market) and 8% (https://www.grandviewresearch.com/press‐release/global‐prebiotics‐market), respectively. Foreseeing this rise, the manufacturers are not just eyeing for newer and efficient ways of upscaling the production (Mano *et al*., 2018) but are also firing up the research and development sector for carving innovations as per the consumer demand. These innovations are not merely confined to the search of better candidates (like *Faecalibacterium prausnitzii*, *Eubacterium* spp., *Bacteroides* spp. and more) (Cunningham *et al*., 2021) but are also paving way for niche markets that are developing age, gender, demography specific probiotics and prebiotics. The latter are also being increasingly used in diverse applications ranging from infant feed to their use as an alternative to sugar or for texture enhancement in food and beverage industries to even animal feeds as a replacement of antibiotics, growth‐promotion and health enhancer (Cunningham *et al*., 2021) for raising the dairy production. The interaction of the probiotics with the host and the microbiome is mediated either through the molecular effectors presented on the cell surface or through the metabolites released as cell secretion. These metabolites have multifocal functionalities ranging from facilitating cross‐feeding interactions to altering the microenvironments in the GI tract, producing bacteriocins and other growth inhibiting compounds (Mikelsaar *et al*., 2016) and even promoting competition for the binding sites and nutrients (Cunningham *et al*., 2021).

## Knowledge gaps and future prospects of probiotics

**Fig. 1:**
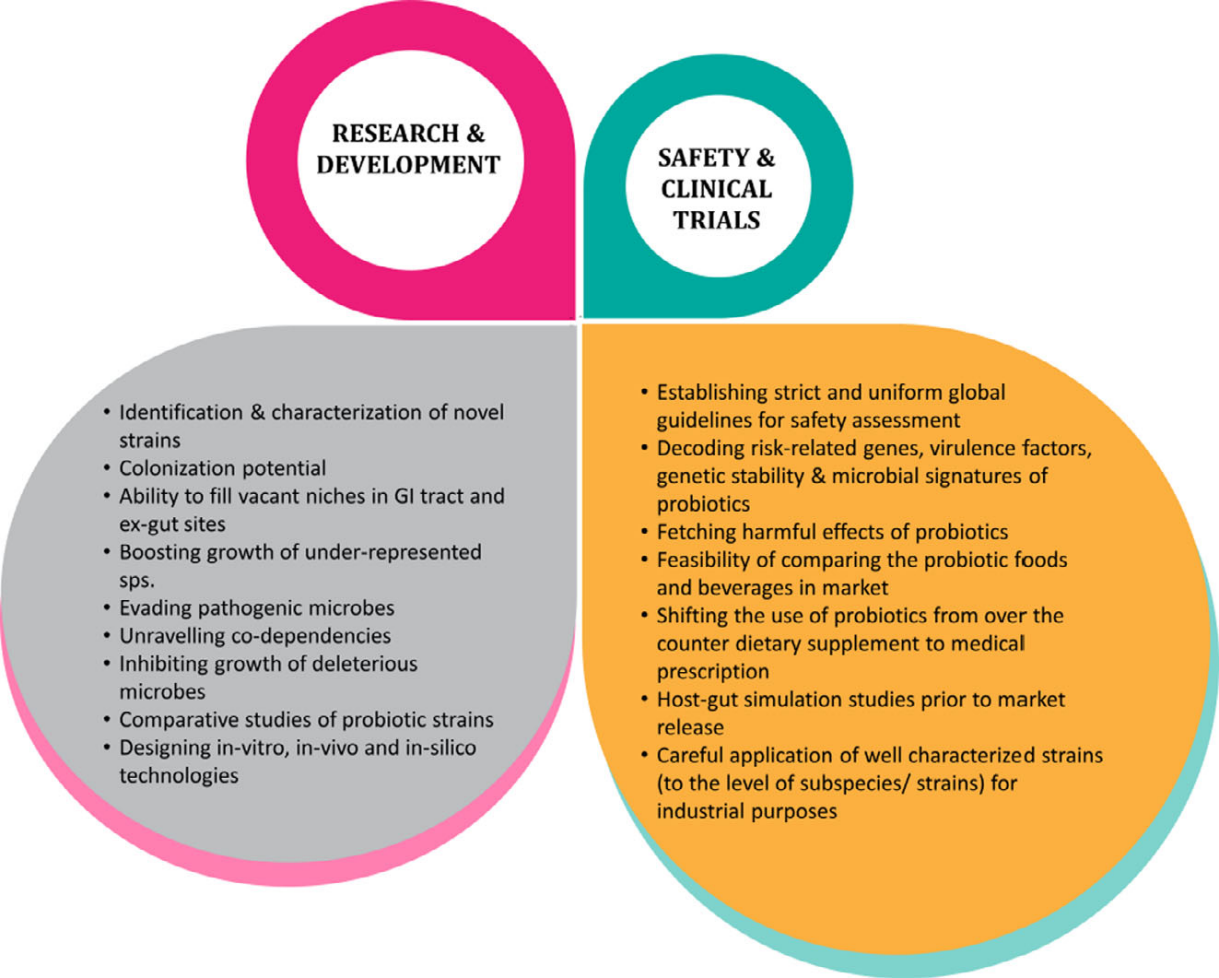
The significance of Research & Development and Safety & Clinical Trials in the future prospect of probiotics.

Despite the investment of tremendous efforts towards deciphering the multi‐faceted characteristics and underlying mechanistic attributes of probiotics, the field exhibits many missing components. There is a pressing need to surface these gaps for unravelling the bigger picture and prospecting the future of probiotics (Fig. 1). These knowledge gaps chiefly require the following investigations:

1. Isolation, identification and characterization of novel strains capable of colonizing vacant microbiome niches of ex‐gut sites besides the GI tract (Gibson *et al*., 2017; Cunningham *et al*., 2021).
2. Harnessing novel probiotics for establishing microbiome replenishment strategies to offer specific health benefits and metabolic outputs (Cunningham *et al*., 2021).
3. Ameliorating probiotics to meet precise host requirement based on the introduction of beneficial microbes to fill the vacant niches, accelerating the growth of under‐represented species or by suppressing the growth of pathogenic microbes either by altering the microenvironment or by producing inhibitory compounds (Langille, 2018).
4. A promising scope for future investigations lies for the issue of host non‐responsiveness to probiotics (Reid *et al*., 2010). It has been well established that the successful impact of probiotic colonization varies from one individual to other due to age, gender (Thushara *et al*., 2016), geographic location, lifestyle, dietary habits and also environmental conditions (Upadrasta and Madempudi, 2016). The future might behold a solution to overcome this variation and developing a strategy that might aptly follow the concept of ‘One shoe fits for all’.
5. Detailed comparative studies to investigate unique, distant and specific effect of every component of a probiotic or its blend are of prime significance. Supplemented with machine learning and artificial intelligence (Cunningham *et al*., 2021), probiotics have a scope of being used in clinical care for safe and targeted delivery of disease specific bioactive compounds (Spacova *et al*., 2020).
6. There is a growing need to evaluate the genetic stability and safety assessment of virulence factors, risk‐related genes and antibiotic resistance of the probiotic microbes. It is of utmost importance to broaden the genetic tool box to monitor the real‐time microbiome and health status of the host (Spacova *et al*., 2020).
7. As the breadth of knowledge in the field of microbiome science is expanding, it is escalating research in many associated and overlapping microbiome‐modulating interventions. These include the rise of Next Generation Probiotics majorly comprising of synbiotics, post‐biotics, biotherapeutics with live microbes, microbial consortia, GMOs and also the use of microbial signatures for precision medicine interventions and personalized probiotics (Cunningham *et al*., 2021).

In the present scenario where the field of probiotics is expanding with no bounds and leaps, there still exist several voids in terms of absence of the appropriate tool set, unavailability of a legal framework to address the ethical issues and use of GMOs (Forssten *et al*., 2020; Grumet and Tromp, 2020; Spacova *et al*., 2020). It is thus imperative to drive efforts towards uniform quality assurance. Future consumption of probiotics necessitates the availability of standardized assessment and certification rules to ensure the use of strains that are taxonomically characterized to the level of subspecies or strain rather than to just the genus (Binda *et al*., 2020). Though many nations across the globe have devised stringent regulatory frameworks, the evidence for the claimed influence on health (Cunningham *et al*., 2021) still remains quite scarce and inadequate. In a recent study, the American Gastroenterological Association (AGA) tried to stitch the role of probiotics in the management of gastrointestinal disorders. Based on their systematic analyses on the documented use of probiotics, it was reported that the clinical trials are either missing or are inadequate in clearly relating the beneficial impact of probiotics to the species level particularly. In absence of any discrete guidelines for the testing of a probiotic product under clinical trials, it has become even more difficult to compare and assess the same probiotic manufactured and marketed under different labels (Preidis *et al*., 2020). Additionally, reports on the deleterious effects of probiotics are increasingly missing across the published studies. In light of insufficient information pertaining to probiotics, they have been taken off from the current clinical dietary guidelines for the treatment of gastrointestinal disorders (Preidis *et al*., 2020). Only upon presentation of concrete evidence based on stringent clinical trials within the limits of guidelines would call for inclusion in future.

Hence, one may speculate that with an enormous history of probiotic consumption for several centuries and a wealth of probiotic and microbiome research till date, a large knowledge gap pertaining to their specific role, ability to colonize the GI tract, co‐dependencies based on species/strain and site of action still persists. Also, a little is known about their exact capabilities in keeping away infections and diseases. In absence of strong evidences in attesting a beneficial role of probiotics in countering pathogenic microbes, the success of probiotics still stays incompletely portrayed. Moreover, it is becoming increasingly evident that the beneficial role of microbes cannot be extrapolated for all the probiotic formulations (Preidis *et al*., 2020). The sale of over the counter probiotics as dietary supplements thus require serious interventions until this prevailing knowledge gap gets filled as an outcome of dedicated research and emerging technologies. Until then, the future of probiotics seems sufficiently unclear.

## Frontiers of Space microbiome in the next decade

Humans are destined to explore space but astronauts can contract a variety of diseases during these long space missions. ‘The space exposome’ comprises stressors like microgravity, fluid changes, interstellar cosmic radiation beyond the Earth's Van Allen Belt, sleep deprivation with altered circadian patterns and extended isolation and confinement. (Crucian *et al*., 2018). Space exposome together with the sterility of the spacecraft, diet in space and environmental stresses may cause changes in the intestinal microbiota, impairing immunity and putting astronauts at risk of illness. The increased virulence of pathogens in microgravity adds to this concern and can make extra‐terrestrial travel impossible (Saei and Barzegari, 2012). As a result, the provision of effective life support systems during long‐term missions into space has attracted a lot of interest in space medicine research. Normally, we are continuously replenishing our microbiomes with a new set of species that help us to keep a diverse and balanced microbiome (Costello *et al*., 2009; David *et al*., 2014; Lloyd‐Price and Abu‐Ali, 2016). Thus, under natural earth conditions, there exists a symbiotic homeostasis between the host, microbial commensals and several potentially pathogenic bacteria in the microbiome (Saei and Barzegari, 2012). However, astronauts do not receive the same replenishment of microbes on a space flight as they do on earth. Stressful environments, such as those encountered on a space flight, including cosmic radiation (Packey and Ciorba, 2010) and microgravity (Nickerson *et al*., 2004) have been shown to promote microbial dysbiosis and changes in bacterial physiology. Previous research on culturable commensal and opportunistic pathogenic bacteria in real or simulated microgravity has indicated that space travel may trigger functional changes in the crew microbiome, such as bacterial virulence, antibiotic resistance and biofilm formation (Ciferri *et al*., 1986; Benoit *et al*., 2006; Klaus and Howard, 2006; Shao *et al*., 2017) Furthermore, culture‐based methodologies on astronaut samples obtained before and after space missions have discovered changes in the microbial composition of the dental, nasal and intestinal microbiota (Brown *et al*., 1976; Decelle and Taylor, 1976; Lencner *et al*., 1984; Lizko and Silov, 1984). With the announcement of Mars rover missions 2020 by United Arab Emirates, China and the United States of America (Witze and Mallapaty, 2020), long‐duration manned missions to Mars and beyond are expected in the coming years. Hence, it becomes imperative to conduct extensive microbiome research to better maintain microbiome homeostasis and astronaut health on future long‐duration exploration missions.

### Altered membrane properties in response to simulated microgravity (SMG)

In space, all organisms and even cells would respond to changes in microgravity (Demontis *et al*., 2017). Bacteria thicken their cell walls in response to the absence of this ubiquitous force, resulting in an increase in the minimum inhibitory concentration (MIC) of antibiotics (Tixador et al., 1985b,1985a). Drug resistance developing this way during a space flight, could pose a serious threat for the entire crew, as it is difficult to treat (Saei and Barzegari, 2012). For example, in *E. coli* grown aboard, for both colistin and kanamycin, increased antibiotic resistance (or MIC) was observed as compared with the MIC on the ground (Tixador et al., 1985b,1985a). A similar increase in the MIC of oxacillin, erythromycin and chloramphenicol was reported in *Staphylococcus aureus* (Tixador et al., 1985b,1985a). The emergence of drug resistance during space missions is a matter of concern, and it is possible that drug resistance is aided by bacterial mutations, which are much more likely during long‐term space flights (Fukuda *et al*., 2000). Microgravity was seen to increase bacterial growth as well, relative to terrestrial controls (Kacena *et al*., 1999), and bacteria also exhibit elevated virulence under such simulated reduced gravitational conditions (SMG). This is demonstrated by increased animal death after injection of *Klebsiella pneumoniae* in simulated weightlessness, as opposed to controls (Belay *et al*., 2002; Kirkpatrick *et al*., 2020). *Salmonella typhimurium* cultures were also more virulent and were recovered in greater numbers from the spleen and liver of the tested animals (Wilson *et al*., 2007). *Salmonella* grown under microgravity were also found to be less susceptible to acid stress and macrophage death (Wilson *et al*., 2007). These findings coincide with those of increased biofilm formation and antibiotic resistance in *E. coli* (Lynch *et al*., 2006) and *Pseudomonas aeruginosa* (Crabbe *et al*., 2008, 2011) under microgravity conditions. Under SMG conditions, enterotoxigenic *E. coli* produces a significant amount of heat‐labile enterotoxin (Chopra *et al*., 2006). Likewise, *Salmonella* enterica *Typhimurium* cultured aboard space shuttle mission exhibited increased virulence in mice, increased survival within a macrophage cell line and increased extracellular matrix accumulation consistent with a biofilm, as compared with appropriate ground controls (Nickerson *et al*., 2000; Wilson *et al*., 2002). Not only bacteria, but the fungi *Candida albicans* also exhibited increased filamentation, biofilm formation and increased resistance to anti‐fungal agent amphotericin B under SMG (Searles *et al*., 2011). Biofilms’ properties make them a significant risk factor in long‐duration space flights, as they increase the risk and severity of infection. Bacterial biofilms have been related to difficult‐to‐treat diseases such as endocarditis, cystitis and bacterial otitis due to their properties of increased tolerance to not only antibiotics but also oxidative, pH and osmolarity stresses (Costerton *et al*., 1987; Fux *et al*., 2005).

### Microbiome dysbiosis in response to simulated microgravity

Omics‐based methods have exploded in popularity in recent years, not only for detecting microbiota dysbiosis but also for testing technologies from a health‐promoting standpoint during missions (Turroni *et al*., 2020). The changes in the human commensal microbiome in response to changes in the environment and behaviour are much more significant than the isolated microbial changes (in gene expression and cellular anatomy). Samples were analysed from the eyes, throat, urine and faeces of the six crew members both before and after the Apollo missions as early as in the 1970s (Taylor and Henney, 1973). In the nasal flora, increase was observed both in the number of non‐pathogenic bacteria and opportunistic pathogens (Nefedov *et al*., 1971; Taylor *et al*., 1973). One another study discusses a significant decrease in the numbers of *Bifidobacterium* and *Lactobacilli*, as well as a significant increase in the numbers of *E. coli* and *Enterobacteria* during the flight's preparation phase. This might be attributed to psychological stress before take‐off (Lizko *et al*., 1984). Jiang *et al*. (2019) flew mice for 37 days on the International Space Station (ISS) and 13 days on Space Shuttle Atlantis STS‐135. The experiments corroborated previous results by Lencner *et al*. (1984) (Lencner *et al*., 1984). The microbial community's abundance did not change, but the community composition did, with a higher Firmicutes‐to‐Bacteroidetes ratio (Jiang *et al*., 2019). Not surprisingly, these findings in mice are consistent with those of a recent study involving twin astronauts (Garrett‐Bakelman *et al*., 2019). Two identical twins were studied, one of whom spent a year on the International Space Station and the other on Earth. Before, during and after a year, the physiology, memory abilities and genetic material, among other aspects, were all monitored. While prolonged space travel affected many, if not all, physiologic functions, most of them returned to normal levels after returning to terrestrial gravity on Earth. Some changes persisted, such as decreased gene expression, increased DNA damage and the number of short telomeres, as well as diminished cognitive function. The Firmicutes‐to‐Bacteroidetes ratio increased during spaceflight but did not persist after returning to Earth (Garrett‐Bakelman *et al*., 2019). Low levels of metabolites including 3‐indole propionic acid (having anti‐inflammatory properties) were also reported. Indoles are produced by commensal bacteria from tryptophan and are involved in immune system modulation.

Microbes associated with astronauts and spacecraft have been a subject of great interest. The microbiome of the surfaces inside the ISS is affected and resembles that of the crew's skin (Voorhies *et al*., 2019; Avila‐Herrera *et al*., 2020). Mission HI‐SEAS IV (Hawaii Space Exploration Analog and Simulation IV), an investigation into microbial transition between crew and habitat over the course of a year in 2021 revealed substantial variations in microbial diversity, abundance and composition between samples of the built environment and its crew (Mahnert *et al*., 2021). Within the first 200 days, a regular transfer of the indicator species *Methanobrevibacter* between crew members had a significant impact on skin microbiome dynamics. The spread of AMR in the habitat was tracked using quantitative data (Mahnert *et al*., 2021). Most skin‐associated bacteria, such *as Staphylococcus aureus, Brevundimonas, Kocuria, Propionibacterium, Streptococcus, Kytococcus* and *Dermacoccacae*, could be easily traced in the habitat, and were most commonly shared with the desk space, and were more likely transferred between crew members who had near physical contact with each other. Alterations in the skin microbiome were also discovered, which may explain why astronauts in space have such a high level of skin rashes/hypersensitivity episodes. Further research is essential in order to comprehend negative outcomes in a possible base on the Moon or Mars. Similarly, the Astronaut Microbiome Project is a major research study currently underway at the International Space Station that aims to use culture‐independent methods to study the microbiome of astronauts, surfaces and water (Voorhies *et al*., 2019).

Attempts have been made to identify early warning symptoms of any complications that may arise during space explorations. The temporal dynamics of six crew members were analysed in a 520‐day simulation experiment in Moscow, Russia (MARS500) (Turroni *et al*., 2017). The shifts were investigated on psychological, intestinal and immune system levels. After about a year of confinement, the study revealed increased relative abundance of *Bacteroides* spp. in the early stages of the mission and decreased proportions of some short‐chain fatty acid (SCFA) producers, especially *Faecalibacterium prausnitzii*. The MARS500 mission was the world's first long‐term simulation of a crewed return flight to Mars. In another experiment called MICHA (MIcrobial ecology of Confined Habitats and humAn health), in 2017, the microbiology of the system, that is crewed habitat, was investigated further in order to identify hotspots for dispersal and aggregation of stress‐resistant and potentially pathogenic crew microorganisms (Schwendner *et al*., 2017). Hotspots for microbial aggregation have been established in areas of high human activity. On different surfaces, cultivation assays showed a microbial population dominated by *Staphylococcus* and *Bacillus*. A recent simulation experiment known as BLSS (Bioregenerative life‐support system) was performed in a confined, self‐sustaining artificial ecosystem to biologically regenerate O2, food, water and other essential living necessities in order to maintain a eubiotic gut microbiome. The crew adhered to a strict regimen that included several hours of daily plant contact and a high‐fibre, plant‐rich diet. The microbiome became more diverse and rich, with a rise in the relative abundance of certain SCFA producers and a decrease in the proportions of potential pathogens. Thus, in long‐duration space flights, maintaining a high‐fibre diet is important, according to this report (Hao *et al*., 2018; Chen and Zhou, 2021).

### The future of space microbiome

If we were to imagine future existence of life on different planets within our solar system, microbes will act as the pioneers for habitat seeding. Some commercial companies and NASA have already stated their plans to colonize Mars or some another planetary body capable of supporting life (Lopez and Peixoto, 2019). For this to become a possibility, long‐term space exploration missions will be needed, but this is currently impossible due to a variety of factors. In this review, we have discussed one of those restricting factors, that is, the astronauts' ‘altered microbiome’. More research is needed to better understand and describe the complex relationship between the host and the gut microbiome during and after the spaceflight. Such research will be expected to contribute to the formation of strategies that would aid in the adaptation of microbial communities to their spaceflight‐related environment (Jiang *et al*., 2019). By preventing any harm and conferring benefits to the host, such interventions would facilitate long‐term space flight.

The existing research raises some important questions about microbiomes in space. We need to know how diverse a microbiome needs to be during space travel and what its optimal composition should be, both within the host and on ISS surfaces, for future missions. Another important parameter that needs to be investigated is how such a microbial composition can be stabilized during travel and returned to levels that on Earth once the mission is completed. As this aspect deals with astronaut health and disease, the solutions to these main parameters will be important in preparing future manned long‐term missions into space and other planetary bodies (Mahnert *et al*., 2021).

1. In order to safeguard astronauts' health and meet their nutritional needs in space, future missions will need to develop probiotics tailored for each crew member. The healthy bacteria individually selected for each microbiome could be consumed during long‐term missions to replenish or mimic the microbiome as it exists on earth. A balanced gut microbiome profile is low in pathogens and high in members capable of developing SCFAs (Turroni *et al*., 2020). The available studies thus support the administration of SCFA‐producing next‐generation probiotics such as *Akkermansiamuciniphila*, *Faecalibacterium*, and *Roseburia* (O'Toole and Marchesi, 2017). However, more investigation and studies are needed before releasing such products (Turroni *et al*., 2020).
2. The alteration of the GI microbiota in space may be a major contributor to the crew members' immune system dysregulation during space flights. As a result, diet‐based treatments (such as a high‐fibre diet) that support a healthy microbiome composition can be used to manage these dysregulations (Turnbaugh *et al*., 2009). Increasing the prebiotic content of the space diet, in conjunction with the research on hydroponic gardens may be a lucrative alternative.
3. Since astronauts will need to bring food with them for the entire duration of the mission, the food's composition and shelf life will be severely limited (Cooper and Douglas, 2011). Thus, for it to be a viable choice as a space food, durability, long‐term storage and the nature of the microbes to be used as food will require comprehensive studies. Because the ISS is orbited within the Van Allen Radiation Belt, astronauts are partially shielded from the harmful effects of GCR (Galactic Cosmic Radiation). However, there is still a lack of knowledge about the effects of these low‐dose radiations on their microbiomes. Most microbes are radiation sensitive, and studies conducted on earth to see how high doses of radiation affect the microbiome have noted significant damage to it (Packey and Ciorba, 2010; Lam *et al*., 2012). As a result, GCR is expected to harm the majority of microbes on the ISS, not just those in probiotic packages, but also those inside and on the surface of astronauts and the ISS (https://doi.org/10.3389/fspas.2016.00023).
4. The use of probiotics in the mitigation of musculoskeletal system issues has also been tested. They can do this by controlling the synthesis of vitamins and co‐enzymes involved in the formation of bone matrix. Microbiota‐produced SCFAs are also involved in mineral absorption in the intestine, such as calcium (Collins *et al*., 2017). Increased SCFA production has been attributed to a greater bone density in animal models in some studies (Chen *et al*., 2017; Ohlsson *et al*., 2017; Ibanez *et al*., 2019).
5. The health of astronauts and the performance of every space mission may be compromised if their neurocognitive and psychomotor abilities deteriorate (De la Torre, 2014). Given the well‐known ‘gut‐brain axis’, strategies aimed at promoting a balanced gut microbiome can also aid in reducing the negative effects on the brain and behaviour (De Palma *et al*., 2014). The gut microbiome produces both neuroactive and neurotoxic metabolites, such as SCFAs, tryptophan metabolites, neurotransmitters like GABA and nitric oxide, and neurotoxic metabolites like D‐lactic acid and ammonia (Galland, 2014). Despite the fact that these studies are mainly focused on murine models, there is evidence that probiotics can help with mental health when appropriate strains are chosen (Romijn and Rucklidge, 2015; Reis and Ilardi, 2018). This is most likely accomplished by controlling the development and release of neuroactive substances (Turroni *et al*., 2020).

Once the researchers have a clearer understanding of the changes in microbiome as a result of the microgravity setting, appropriate recommendations to include prebiotics, probiotics and post‐biotics to address pathological effects in astronauts can be made.

## The next pandemic we may expect

The entire world took flabbergast by the devastating Covid‐19 pandemic. But epidemiologists and experts from different parts of the world had long back set a forecast that we were setting ourselves up for a global pandemic (Taylor and Latham, 2001; Lau *et al*., 2005). A common fact that can be drawn out of these newly emerging diseases is their animal origin. As a matter of fact, 75% of newly emerging diseases are zoonotic in nature (Taylor *et al*., 2001). Even, Covid‐19 finds its likely origin from bats, which serve as reservoir hosts for its progenitor (Andersen *et al*., 2020). The anthropogenic effect on the global climate, encroachment on wildlife habitats and global travel has facilitated and furthered the spread of animal‐borne diseases. We have already set up a hot bed for more pandemics to set pace by contributing towards urbanization, overpopulation and global trade. In the section that follows, we have tried to scrutinize the current potential microbial pathogens that pose the utmost pandemic threat, their possible causes and the ongoing research that aims to mitigate their impact.

### Ebola virus disease (EVD)

Ebola virus, first recognized in 1976 near the Ebola River in modern day Democratic Republic of the Congo (DRC) is an emerging and re‐emerging zoonosis (Dhama *et al*., 2015). Akin to many zoonotic viruses, Ebola has originated from fruit bats of the *Pteropodidae* family (Dhama *et al*., 2015). Since then, there have been several outbreaks in African countries with the biggest eruption in 2014–2016 in three West African countries: Liberia, Guinea and Sierra Leone (Valle *et al*., 2021). It caused at least 11 000 and > 2200 fatalities in the recent outbreaks in West Africa (2013–2016) and in the DRC during 2018–2020, respectively (Valle *et al*., 2021). All the four life‐threatening Ebola viral strains are endemic to Africa, mainly spread through a direct exchange of bodily fluids, and through respiratory droplets, whereas the fifth strain, Reston virus (RESTV), is pathogenic to animals but non‐pathogenic in humans (Cantoni *et al*., 2016). Despite the availability of two licensed vaccines, there is a growing concern regarding ineffectiveness of vaccine against RESTV or another strain of Ebola virus, which could mutate and spread through imported livestock or other sources.

### Nipah virus

Nipah virus first spread in 1998 during a large outburst of encephalitis and respiratory disease in Singapore and Malaysia that caused 276 cases of Encephalitis with 106 fatalities (Ang and Lim, 2018). The disease is now circulating in South‐East Asia, with outbreaks in Bangladesh, India, Malaysia and Singapore and has been listed in the top 10 priority diseases notified by WHO that behold the prospect of triggering a pandemic in near future (Epstein *et al*., 2020). Originated from fruit bats of *Pteropodidae* family, the major concern associated with the infection is the high mortality rate (up to 40–70%) depending on where the outbreak occurs (Epstein *et al*., 2020). There are endless opportunities for the virus to spread from bats to other animals and to humans, since fruit bats live in trees that may be in close proximity to markets, schools, places of worship and tourist spots. There are quite a few reasons which makes Nipah virus so threatening. The long incubation period of virus, as long as 45 days, higher risk of infection to wide range of animals, spill over from bats to animals and humans and their fast spread either through direct contact or by consuming contaminated food (Singh *et al*., 2019). The more dreadful is the unavailability of vaccine or treatment for this deadly viral disease. Scientists still believe that encroachment and overdevelopment on bat habitat is making another spread‐out likely in coming future.

### Chikungunya fever (CHIKF)

Chikungunya is caused by an arbovirus chikungunya virus (CHIKV) that belongs to alphavirus of *Togaviridae* family and was first isolated in 1953 in Tanzania. The infection is transmitted by *Aedes* mosquitoes (Translational Research Consortia for Chikungunya Virus in, 2021). In particular, it is a tropical disease, but its outbreaks are relatively uncommon due to its geographical restriction. The excruciating disease causes unbearable fever, headache, joint pain as well as joint swelling, muscle pain, fatigue, nausea and rash (Cunha and Trinta, 2017). In the year 2006, India reported 14 000 000 cases of chikungunya in which *Aedes aegypti* was the presumed vector (Pialoux *et al*., 2007). An outbreak of chikungunya spread across Latin America infected nearly 800 000 people across 31 countries in 2013 (Fischer *et al*., 2014). The global climate change may offer a new habitat for mosquitoes in America and parts of Europe, the unaffected part of the world where chikungunya could not spread will become another endemic region in near future. In a nutshell, the deadly combination of globalization and climate change makes chikungunya more prone to spread globally and there is an ever growing prospect of another pandemic hitting the world if the current situation remains unaltered.

### H5N1 and H7N9 influenza

In recent years, there has been an emergence of several novel viruses from different parts of the world that predominantly manifest as respiratory tract diseases in human beings. For instance, the surge of highly pathogenic avian influenza (HPAI) is pervasive due to its zoonotic hosts including birds, pigs and others that serve as reservoirs for the virus and also owing to their increased probability of transmitting the infection to humans. HPAI H5N1 virus was first detected in a goose farm in southern China in the year 1996 and was reported in humans in 1997 in Hong Kong (Chan, 2002; Wan, 2012). The first reports of HPAI H7N9 came out from Eastern China in 2013 (Gautret *et al*., 2014), whereas Shanghai and Anhui were the first two cities to report human cases infection (Wu and Xiao, 2020). With three major waves of outbreaks of the H7N9 virus in past from mainland China, all the occurrences followed a similar seasonal trend (Tanner and Toth, 2015). Before COVID‐19, it was widely assumed that the next pandemic would be caused by influenza. Due to availability of seasonal influenza vaccines, the virus is widely recognized to be low risk by the common masses, to a degree, with those most at risk being infants and older people. Even so, the risk is far above the ground particularly with two subtypes, H5N1 and H7N9 and the probable more variants, which has not been reported yet.

### Zika flavivirus

The unexpected and far‐reaching outbreak of coronavirus (SARS‐CoV‐2) has shifted the limelight to another developing viral threat, the Zika flavivirus. It is transmitted to humans mostly by *Aedes aegypti* and *Aedes albopictus* mosquitoes and upon sexual contact with infected persons (Kassavetis *et al*., 2016). Both *A. albopictus* and *A. aegypti* can transmit diseases like chikungunya, dengue and yellow fever except for malaria. The virus was first identified in a febrile Rhesus monkey in the Zika Forest of Uganda in the year 1947 (Ribeiro and Kitron, 2016). Since 2015, Zika is spreading with an alarming pace; with reported outbreaks in 87 countries (Rather *et al*., 2017). Natural incidents like hurricanes have also been well‐known to move mosquitoes from one continent to the next. But the real grounds are provided by humans, who unwittingly transport these small disease vectors such as mosquitoes faster and farther than any storm with their ever‐growing ease of transport network like trucks, ships and airplanes. Undeniably, globalization is jeopardizing to unleash the next pandemic certainly revealed by the overarching burden of Zika virus.

## Conclusions

With the advancements in microscopy and sequencing, the next 15 years will surely put light on microbial dark matter. Presently, the uncharacterized diversity constitutes almost 50% of the total microbiota in metagenomics studies. The inputs from omics approaches are now being utilized to devise and improvise culturing methods for culturing the unculturable. The next decade shall witness improvements in microbiology and microbiome techniques leading to the characterization of novel microbes and their products that can be beneficial for mankind. Microbiology literacy has a key role to play in reducing the spread of AMR. Further, newer methods based on the information from molecular microbiology and microbiome are paving way for a futuristic scenario of personalized medicine. The mechanisms of drug resistance have helped us to cope up with pathogenic bacteria and the newer approaches like phage therapy, quorum quenchers, repurposing of existing drugs have the potential to be developed as an alternative to the traditional antibiotics. The human gut microbiome has also an important role to play in formulating personalized medicine as many diseases have been linked with dysbiosis of microflora and restoring the normal diversity can be used to lower the impact and onset of respective diseases. Probiotics are considered to have an important role in restoring the gut microflora of humans. Studies in the future will be more focused on developing newer probiotics, checking their efficacy and their side effects that are still unknown. Microbiological research will progress on the space frontier in complement to the advancements on earth. The next decade will surely witness extensive microbiome research to safeguard astronaut health and better maintain microbiome homeostasis. Given the rapid pace of population growth, human migration, rapid global travel and climate change can all hasten the spread of infectious diseases. Owing to the way people encroach on animal habitats, it is increasingly predictable that zoonotic diseases spill over from animals to humans and will cause future pandemics. Certainly, the Covid 19 pandemic was not the first to ruin the world and it will not be the last. In the end, it is imperative to understand the rising dominance of microbiology as a saviour against the newer pathogenic microbes and subsequent pandemics.

## Funding information

No funding information provided.

## Conflict of interest

The authors declare no conflict of interest.
